# Bone Quality Beyond DXA in People Living with HIV: A Systematic Review of HR-pQCT, TBS, Microindentation, and Vertebral Fractures

**DOI:** 10.3390/jcm14217669

**Published:** 2025-10-29

**Authors:** David Vladut Razvan, Ovidiu Rosca, Felix Bratosin, Vlad Predescu, Silviu Valentin Vlad, Adrian Vlad

**Affiliations:** 1 Doctoral School, Faculty of Medicine, Victor Babes University of Medicine and Pharmacy, 300041 Timisoara, Romania; vladut-razvan.david@umft.ro; 2 Methodological and Infectious Diseases Research Center, Department of Infectious Diseases, Victor Babes University of Medicine and Pharmacy, 300041 Timisoara, Romania; ovidiu.rosca@umft.ro (O.R.); felix.bratosin@umft.ro (F.B.); 3 Orthopaedics and Traumatology Department, Ponderas Academic Hospital, 014142 Bucharest, Romania; 4 Department of Surgery, Faculty of Medicine, University of Oradea, 410073 Oradea, Romania; 5 Centre for Molecular Research in Nephrology and Vascular Disease, Faculty of Medicine, Victor Babes University of Medicine and Pharmacy, 300041 Timisoara, Romania; vlad.adrian@umft.ro; 6 Department of Internal Medicine II, Division of Diabetes, Nutrition and Metabolic Diseases, Victor Babes University of Medicine and Pharmacy, 300041 Timisoara, Romania

**Keywords:** bone density, finite element analysis, HIV infections, magnetic resonance imaging, microindentation, osteoporosis, quantitative computed tomography, trabecular bone score, vertebral fractures

## Abstract

**Background and Objectives**: People living with HIV (PLWH) have excess fragility fractures not fully explained by areal DXA. We reviewed bone “quality” in PLWH—microarchitecture, estimated strength, tissue-level properties—and vertebral fractures (VFs). **Methods**: PRISMA-conform systematic review (2000–2025) of randomized, cohort, and cross-sectional studies assessing HR-pQCT (±finite-element analysis), trabecular bone score (TBS), impact microindentation (BMSi), femoral QCT/MRI, and VF imaging (DXA-VFA or radiography). Risk of bias used ROBINS-I (non-randomized) and RoB 2 (randomized/switch). No meta-analysis was performed due to clinical/methodological heterogeneity; evidence was synthesized narratively per SWiM. **Results**: Fourteen studies met criteria. HR-pQCT showed cortical/trabecular deficits with lower finite-element–estimated strength in PLWH. BMSi was 3–4 units lower; it declined after ART initiation but improved after TDF→TAF switch. TBS was modestly lower and reclassified risk when BMD was non-osteoporotic. VF prevalence was 12–25% and frequently occurred at non-osteoporotic BMD. Signals aligned with modifiable risks (smoking, glucocorticoids) and specific ART exposures. **Conclusions**: Beyond DXA, PLWH exhibit quantifiable decrements in microarchitecture, estimated strength, and tissue-level properties alongside a meaningful VF burden. TBS and VFA are pragmatic, scalable adjuncts to refine risk; HR-pQCT/BMSi add mechanistic value in research/tertiary settings. Prospective studies linking these metrics to incident fractures are warranted.

## 1. Introduction

Fracture burden remains disproportionately high among people living with HIV (PLWH), even in the era of durable virologic suppression, and is only partially explained by areal bone mineral density (BMD) on DXA [1]. Contemporary guidance emphasizes systematic screening and risk modification in HIV care, reflecting both HIV-related and treatment-related skeletal liabilities [2]. In randomized evidence from the START BMD substudy, immediate antiretroviral therapy (ART) initiation accelerated BMD loss at hip and spine versus deferred therapy, highlighting a treatment-onset effect superimposed on background vulnerability [3]. Longer-term follow-up indicates that this early decline attenuates after the first 1–2 years, but does not eliminate the elevated fragility signal seen epidemiologically [4].

Therapeutic choices also matter. Multiple switch trials demonstrate skeletal safety advantages when replacing tenofovir disoproxil fumarate (TDF) with tenofovir alafenamide (TAF), with improvements in hip and spine BMD and tubular markers in diverse populations, including older adults and those with renal impairment [5,6,7]. These observations reinforce the need to look beyond areal BMD to capture dimensions of bone quality that may mediate residual fracture risk despite ART optimization.

Microstructure-oriented metrics offer that lens. Trabecular bone score (TBS)—a texture index derived from lumbar DXA—predicts osteoporotic fractures independently of BMD and can refine FRAX probabilities in general populations [8,9]. High-resolution peripheral quantitative CT (HR-pQCT) provides in vivo “virtual biopsy” of distal radius/tibia, quantifying trabecular number, separation, and cortical thickness; prospective cohorts show that HR-pQCT microarchitecture and estimated failure load independently forecast incident fractures beyond DXA [10,11]. Translating these insights into HIV cohorts, emerging data indicate that PLWH exhibit lower TBS than matched controls and that TBS can prognosticate vertebral fractures, suggesting a clinically actionable signal not captured by BMD alone [12].

Guideline frameworks are beginning to acknowledge these nuances. The European AIDS Clinical Society (EACS) v12.0 update integrates broader musculoskeletal risk assessment and supports judicious DXA use alongside ART selections that minimize bone and renal toxicity [13]. Still, a key evidence gap persists: standardized synthesis of HIV-specific findings from advanced phenotyping, TBS, HR-pQCT, femoral QCT/MRI, and tissue-level measures, linking them to fractures and modifiable drivers (TDF/TAF exposure, protease inhibitor duration, inflammation). Moreover, early ART-associated bone loss, well documented across trials, invites evaluation of whether microstructure/quality measures better capture recovery or persistent deficits than BMD alone [14].

Beyond epidemiology, sex and gender shape musculoskeletal biology and orthopedic outcomes through differences in bone/cartilage/ligament physiology, hormonal milieus, injury patterns, and care pathways. A recent narrative overview in orthopedics synthesizes these sex- and gender-specific mechanisms and highlights implications for personalized care—including the needs of sexual and gender minority patients and potential effects of gender-affirming hormones—reinforcing that sex/gender should be prespecified and reported in HIV skeletal phenotyping [11,12,13,14,15].

We hypothesized that PLWH exhibit measurable deficits in cortical and trabecular microarchitecture and in tissue-level mechanical properties beyond those detectable by areal DXA. Our objective was to synthesize modality-specific evidence (HR-pQCT with FE-strength, TBS, BMSi, femoral QCT/MRI, and vertebral fractures) and relate these to ART exposures and conventional risks.

## 2. Materials and Methods

### 2.1. Protocol, Registration, and Reporting Standards

This review was preregistered on the Open Science Framework (OSF vxkau) prior to screening; any protocol deviations (e.g., inclusion of femoral QCT/MRI encountered during scoping) were logged. The review adheres to PRISMA items on title/abstract, rationale, objectives, eligibility, information sources, search, selection, data items, risk of bias, effect measures, synthesis methods, and certainty assessment, and the PRISMA checklist and flow diagram will be provided in the supplements.

### 2.2. Research Question and PICO Statement

The research question was framed to evaluate bone “quality” in people living with HIV (PLWH) beyond areal BMD. The population comprised PLWH of any age, sex, treatment status, or care setting. The index exposures were HIV infection and antiretroviral therapy (ART) characteristics assessed with bone quality modalities beyond areal DXA, namely HR-pQCT with or without finite-element analysis, trabecular bone score (TBS), impact microindentation yielding Bone Material Strength index (BMSi), femoral QCT, and MRI-based microarchitectural or marrow adiposity measures, as well as vertebral fracture imaging by VFA or standard radiography. Comparators were HIV-negative controls, within-person pre/post contrasts around ART initiation or drug switches (e.g., TDF to TAF), or HIV subgroups such as immunologic responders versus non-responders. Primary outcomes were microarchitectural and material properties, including cortical thickness, trabecular number and separation, volumetric BMD, estimated failure load, TBS, BMSi, and femoral QCT/MRI metrics and marrow adiposity; secondary outcomes were prevalent or incident fragility fractures—especially vertebral fractures—and associations with ART classes, inflammatory indices, immune recovery, and conventional DXA where co-reported.

### 2.3. Eligibility Criteria

Eligible designs included randomized or quasi-experimental trials, prospective or retrospective cohorts, and cross-sectional studies with original quantitative data. We excluded case reports, case series with fewer than 10 participants, narrative reviews, editorials, and conference abstracts without extractable data. Studies had to include PLWH and report at least one bone quality endpoint beyond areal DXA (DXA-only studies without TBS were excluded because our aim was to synthesize quality-oriented metrics beyond areal BMD). No language restrictions were imposed, the time window spanned 1 January 2000 through 1 August 2025, and non-English full texts were translated when feasible. Full-text exclusions were recorded with explicit reasons, including absence of a bone quality endpoint, insufficient data, or overlap with a more complete publication. Vertebral fracture (VF) studies using VFA (DXA-based) were synthesized separately from standard radiography; definitions and thresholds were recorded.

### 2.4. Information Sources

Primary information sources were PubMed/MEDLINE, Web of Science Core Collection (Science Citation Index Expanded, Social Sciences Citation Index, Emerging Sources Citation Index), and Scopus. To enhance completeness, we performed backward citation chasing of all included articles, forward citation tracking in Google Scholar, hand-searched key journals in HIV and skeletal research (AIDS, HIV Medicine, JAIDS, Journal of Bone and Mineral Research, Bone, Osteoporosis International), and screened trial registries (ClinicalTrials.gov and EU-CTR) for relevant completed studies with published bone quality endpoints. Search strategies were PRESS-checked to balance sensitivity and specificity across platforms.

### 2.5. Search Strategy

Searches combined controlled vocabulary and free-text terms for HIV and bone quality modalities, limiting only by publication date and excluding non-human studies where supported. In PubMed/MEDLINE, we used the following string executed on 23 September 2025: “(“HIV”[Mesh] OR “HIV Infections”[Mesh] OR HIV[tiab] OR “human immunodeficiency virus”[tiab]) AND (“high-resolution peripheral quantitative computed tomography”[tiab] OR HR-pQCT[tiab] OR “trabecular bone score”[tiab] OR TBS[tiab] OR microindentation[tiab] OR “bone material strength”[tiab] OR BMSi[tiab] OR “finite element”[tiab] OR FEA[tiab] OR “quantitative computed tomography”[tiab] OR QCT[tiab] OR “magnetic resonance imaging”[tiab] OR MRI[tiab] OR microarchitectur*[tiab] OR micro-architectur*[tiab] OR “bone marrow adiposity”[tiab] OR “marrow fat”[tiab] OR “vertebral fracture assessment”[tiab] OR VFA[tiab]) AND (bone[tiab] OR skeletal[tiab] OR cortical[tiab] OR trabecular[tiab]) NOT (animals[mh] NOT humans[mh]) AND (“1 January 2000”[Date—Publication]: “23 September 2025”[Date—Publication])”. In the Web of Science Core Collection, the topic query was “TS = ((HIV OR “human immunodeficiency virus”) AND (“high-resolution peripheral quantitative computed tomography” OR HR-pQCT OR “trabecular bone score” OR TBS OR microindentation OR “bone material strength” OR BMSi OR “finite element” OR FEA OR “quantitative computed tomography” OR QCT OR “magnetic resonance imaging” OR MRI OR microarchitectur* OR “bone marrow adiposity” OR “marrow fat” OR “vertebral fracture assessment” OR VFA) AND (bone OR skeletal OR cortical OR trabecular))”, with a timespan of 2000–2025, indexes SCI-EXPANDED, SSCI, ESCI, and document type Article. In Scopus, the TITLE-ABS-KEY string was “TITLE-ABS-KEY((HIV OR “human immunodeficiency virus”) AND (“high-resolution peripheral quantitative computed tomography” OR HR-pQCT OR “trabecular bone score” OR TBS OR microindentation OR “bone material strength” OR BMSi OR “finite element” OR FEA OR “quantitative computed tomography” OR QCT OR “magnetic AND resonance AND imaging” OR MRI OR microarchitectur* OR “bone marrow adiposity” OR “marrow fat” OR “vertebral fracture assessment” OR VFA) AND (bone OR skeletal OR cortical OR trabecular)) AND (PUBYEAR > 1999 AND PUBYEAR < 2026) AND DOCTYPE(ar)”. Synonyms and hyphenation variants were tested and retained when they improved recall without excessive noise.

### 2.6. Study Selection and PRISMA Flow

**Figure 1:**
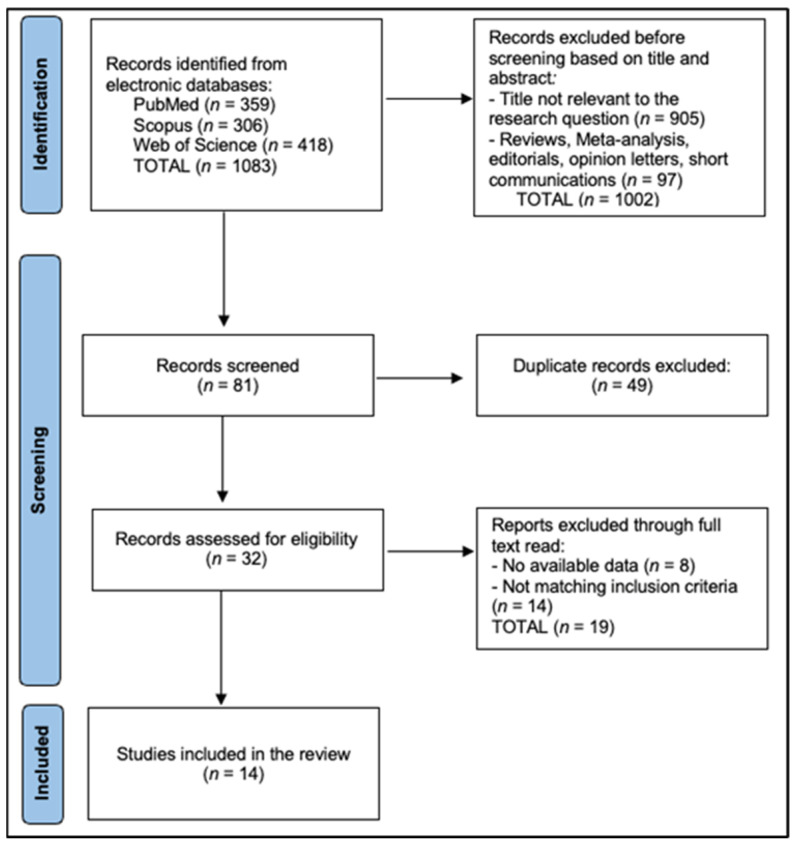
PRISMA Flowchart Diagram.

All records were exported and de-duplicated in EndNote X20 using DOI, title, author, and journal fields with manual verification of near duplicates, then imported to Rayyan QCRI for blinded screening. Two reviewers independently screened titles/abstracts and full texts and independently extracted data (two extractors). Inter-rater agreement was quantified with Cohen’s κ: κ_title/abstract = 0.82 and κ_full-text = 0.86. The reviewers recorded standardized exclusion reasons at full text (DXA-only without TBS, no bone quality endpoint, non-original study, insufficient extractable data, or overlapping cohort superseded elsewhere). Disagreements were resolved by consensus or third-reviewer arbitration. The PRISMA flow diagram is described in Figure 1.

### 2.7. Data Items and Extraction Procedures

Extraction used a calibrated, pilot-tested template capturing study design; setting and country; sample size and participant characteristics; HIV and ART history with emphasis on TDF, TAF, and protease inhibitor exposure; immune and inflammatory markers including nadir and current CD4 counts and viral suppression; bone quality modality and device generation, acquisition sites, and analysis pipelines including finite-element parameters and TBS software version; outcomes including cortical thickness, trabecular number and separation, volumetric BMD, estimated failure load, TBS values, BMSi units, femoral QCT/MRI microstructure and marrow adiposity; vertebral fracture definitions and ascertainment; and statistical adjustments. Corresponding authors were contacted when critical numeric endpoints were missing. When multiple articles reported the same cohort, the most complete dataset or longest follow-up was prioritized.

For HR-pQCT harmonization, we extracted device generation (XtremeCT vs. XtremeCT II), nominal voxel size (measured in µm), in-line vs. manufacturer FE solvers, and segmentation approaches. To enhance comparability, between-group differences were expressed as percent change where possible and synthesized by skeletal site (distal radius vs. tibia) and device generation. Findings mixing generations are marked in tables/captions.

### 2.8. Risk of Bias Assessment

Risk of bias was appraised independently by two reviewers at the outcome level. Non-randomized studies were assessed with ROBINS-I across confounding, selection, exposure classification, deviations, missing data, outcome measurement, and selective reporting. Randomized or randomized-like comparisons (e.g., drug-switch trials) were evaluated with RoB 2. Judgments and rationales were harmonized by discussion with third-reviewer adjudication when needed.

Because of heterogeneity and limited comparable metrics, we applied a narrative synthesis consistent with SWiM guidance. During synthesis, outcomes judged to have serious RoB (ROBINS-I) or high RoB (RoB 2) were down-weighted: effect directions were reported but not allowed to dominate conclusions; where mixed-quality evidence informed a statement, we explicitly flagged certainty and downgraded in GRADE for risk of bias, as presented in Table 1 and Table 2 [16,17,18,19,20,21,22,23,24,25,26,27,28,29]. Across non-randomized comparisons (ROBINS-I), most studies were moderate risk; three were serious for confounding and/or selection; randomized/switch trials were generally low to some concerns (RoB 2). A domain-level summary is provided in Table 1 (ROBINS-I) and Table 2 (RoB 2).

### 2.9. Synthesis Without Meta-Analysis

Due to heterogeneity across study designs, populations, skeletal sites, imaging platforms (HR-pQCT vendors/generations), outcome definitions, and incomplete variance reporting, we prespecified a narrative synthesis per SWiM guidance. We grouped results by modality (HR-pQCT/FE, BMSi, TBS, femoral QCT/MRI, VFs), expressed effects as absolute or percent differences where extractable, and summarized consistency in direction and approximate magnitude. When numeric values were not tabulated, we abstracted effect sizes from figures or text and, where necessary, converted them to percent differences to improve comparability. Where risk of bias was serious/high or inconsistency was marked, we qualified conclusions and reflected this in certainty statements.

## 3. Results

Across 14 studies [16,17,18,19,20,21,22,23,24,25,26,27,28,29] from Europe, North America, and China, cohorts were sizable and modality diverse, enabling triangulation of bone quality in PLWH beyond DXA. HR-pQCT studies consistently enrolled balanced PLWH/control samples: Calmy (92 vs. 95 premenopausal women; radius/tibia) [16], Biver (70 vs. 61 elderly men; radius/tibia) [17], Macdonald (103 vs. 102 women; radius/tibia) [18], and Foreman (103 vs. 77 mixed adults; radius/tibia) [19]. Tissue-level microindentation (BMSi) was examined in cross-sectional and longitudinal Spanish cohorts—Güerri-Fernández (85 vs. 79; tibia) [20], Lerma-Chippirraz (44 starting ART; tibia) [21], Soldado-Folgado 2022 (59 switching TDF→TAF; tibia) [22], Soldado-Folgado 2023 (85 vs. 60; tibia) [23], and Rins-Lozano (82 immunologic responders vs. non-responders; tibia) [24]. Texture-based TBS studies included McGinty (201 PLWH vs. 262 controls; lumbar DXA) [25], Sharma (319 PLWH women vs. 118 controls) [26], and Guan (233 ART-naïve followed 48 weeks post-ART) [27]. Vertebral fracture imaging cohorts (without non-HIV controls) were substantial—Llop (*n* = 199, ≥50 years) [28] and Gazzola (*n* = 194) [29]. ART exposure was long-standing in HR-pQCT cohorts [16,17,18,19], BMSi cohorts captured ART initiation or TDF→TAF switching [21,22,23], and TBS cohorts spanned ART-naïve through long-term therapy [25,26,27], collectively supporting evaluation of modality-specific sensitivity to HIV/ART factors (Table 3).

Microarchitecture and tissue-level quality were consistently worse in PLWH versus controls or reference groups. In HR-pQCT, PLWH showed a lower estimated failure load with trabecular thinning and cortical deficits; Calmy reported ~5–15% decrements across Tb.N, Tb.Sp, and Ct.Th with reduced FEA-derived strength [16], corroborated directionally by Biver in elderly men on long-term ART [17], Macdonald in mid-life women (reduced failure load; cortical deficits linked to TDF history) [18], and Foreman (lower failure load/stiffness associated with PI exposure and smoking) [19]. Tissue-level BMSi was lower in PLWH: Güerri-Fernández 77.2 ± 6.9 vs. 80.6 ± 6.2 (Δ = −3.4; *p* < 0.01) [20]; Soldado-Folgado (2023) 78.4 ± 7.1 vs. 82.0 ± 6.4 (Δ = −3.6; *p* < 0.01) [23]; and immunologic non-responders in Rins-Lozano 76.7 ± 6.3 vs. 80.2 ± 6.1 (Δ = −3.5; *p* = 0.001) [24]. Longitudinally, BMSi declined after ART initiation (−2.1 units by ~6–12 months; *p* = 0.02) [21] but improved after switching from TDF to TAF (+2.5 units at ~6–12 months; *p* < 0.05) [22]. TBS differences were modest but significant: McGinty median 1.349 [1.263–1.436] vs. 1.380 [1.301–1.453] (unadjusted Δ = −0.031; *p* = 0.009; adjusted β for HIV = −0.037; *p* = 0.002) [25]; Sharma observed a 64% higher adjusted prevalence of degraded TBS (<1.35) among HIV-positive women [26]. Vertebral fracture burden remained notable despite often non-osteoporotic BMD—Llop reported ~25% subclinical VFs in older PLWH [28], and Gazzola found 12.4% with predictors including age (aOR 1.09/year) and steroids (aOR 3.64) [29], as seen in Table 4.

**Figure 2:**
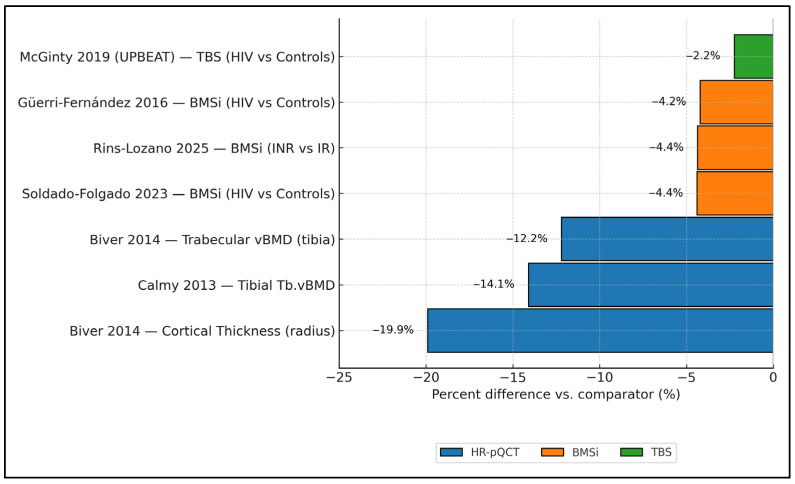
Cross-modality structural impairment in PLWH is expressed as percent differences versus comparators. HR-pQCT results are grouped by skeletal site and device generation (XtremeCT vs. XtremeCT II) where reported; mixed-generation estimates are flagged. Error bars represent reported precision (SD, SE, or 95% CI) when available; otherwise, point estimates only. Data collected from studies included in the final analysis [16,17,20,23,24,25].

Figure 2 synthesizes cross-modality effect magnitudes in PLWH, showing consistently negative percent differences versus comparators. On HR-pQCT, cortical thickness at the radius was ≈−19.9% and tibial trabecular vBMD ≈−12.2% to −14.1% (Biver 2014 [17]; Calmy 2013 [16]). Tissue-level strength (BMSi) was also lower: Güerri-Fernández 2016 [20] showed −3.4% vs. controls (77.2 vs. 80.6 units), Soldado-Folgado 2023 [23] −4.4% (78.4 vs. 82.0), and Rins-Lozano 2025 [24] −4.4% when comparing immunologic non-responders to responders (−3.5 units on an ≈80.2-unit reference). For microarchitecture by TBS, UPBEAT (McGinty 2019 [25]) reported a −2.2% difference (1.349 vs. 1.380). Taken together, impairments are largest for cortical thickness and trabecular density by HR-pQCT, and clearly detectable—although smaller in magnitude—by BMSi and TBS.

Predictor analyses aligned across modalities: Exposure to PIs and current smoking were associated with lower HR-pQCT strength indices (failure load/stiffness) in Foreman [19], while prior TDF were related to worse cortical parameters and reduced failure load in Macdonald [18]. On TBS, HIV status itself carried an adjusted β of −0.037 (*p* = 0.002) in McGinty, with further reductions linked to PI exposure and lower nadir CD4 [25], and HIV-positive women had a 64% higher likelihood of degraded TBS in Sharma [26]. Longitudinally, ART initiation produced early declines with partial recovery by 48 weeks for both TBS and BMD in Guan, and 19.3% had normal BMD but abnormal TBS at baseline, underscoring added diagnostic yield [27]. Tissue-level effects were dynamic: BMSi fell after ART start (−2.1 units; *p* = 0.02) [21] and improved after TDF→TAF switch (+2.5 units; *p* < 0.05) [22]; immunologic non-responders had −3.5 BMSi units vs. responders (*p* = 0.001) [24]; and cross-sectionally PLWH showed −3.6 BMSi units vs. controls (*p* < 0.01) [23]. Fracture-oriented predictors were consistent: older age and steroid exposure increased VF risk—Gazzola aOR 1.09 per year and 3.64 for steroids, with ~70% of VFs occurring at non-osteoporotic BMD [29]—and Llop highlighted ~25% asymptomatic VFs in PLWH over 50 years [28]. Early HR-pQCT studies (Calmy [16]; Biver [17]) already signaled lower Tb.N/Ct.Th and higher Tb.Sp, which are consistent with reduced estimated failure load in PLWH, as described in Table 5.

**Figure 3:**
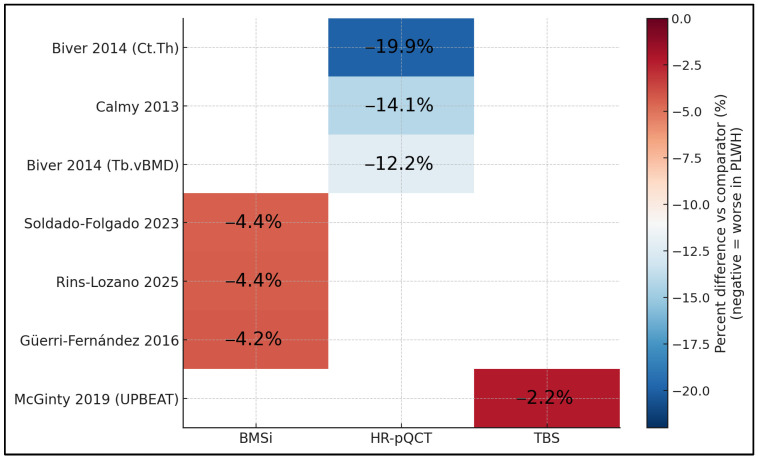
Labeled heatmap of percent differences by study × modality [16,17,20,23,24,25].

Figure 3 compares percent differences vs. comparators across modalities and studies on a common color scale. HR-pQCT shows the largest deficits, with cortical thickness reduced by around −19.9% and trabecular vBMD by −12.2% to −14.1% (Biver 2014 [17]; Calmy 2013 [16]). BMSi results are consistently moderately reduced by about −4.2% to −4.4% across studies (Güerri-Fernández 2016 [20]; Soldado-Folgado 2023 [23]; Rins-Lozano 2025 [24]), while TBS reveals a smaller yet directionally consistent reduction of −2.2% (UPBEAT, McGinty 2019 [25]). The grid makes clear that, regardless of modality, PLWH exhibit uniformly negative deviations, with effect magnitudes greatest for cortical and trabecular metrics captured by HR-pQCT and smaller (but present) for tissue-level (BMSi) and texture-based (TBS) measures.

## 4. Discussion

### 4.1. Summary of Evidence

Our findings of impaired microarchitecture and estimated strength in PLWH align with earlier HR-pQCT work showing disproportionately cortical deficits. In postmenopausal minority women, Yin et al. reported ~11–12% lower tibial cortical thickness/area in HIV vs. controls despite broadly similar trabecular vBMD—an anatomic pattern we also observed at peripheral sites and that plausibly contributes to lower FE-estimated failure load even when areal BMD is not frankly osteoporotic [30]. Methodologically, these strength estimates are robust at the radius/tibia: FE outcomes based on HR-pQCT demonstrate low in vivo precision errors (CV% typically ~1–3% for key parameters), supporting their use for between-group comparisons and longitudinal follow-up in HIV cohorts [30,31,32,33,34]. More recent multimodal imaging at the proximal femur further indicates trabecular-predominant compromise in fracture-prone regions, reinforcing the concept that site-specific quality losses may not be captured by DXA alone [35,36,37,38,39]. Where serious/high RoB or inconsistency arose, we refrained from strength-of-effect language and downgraded certainty accordingly. As a result, overall certainty for vertebral fracture prevalence was low to moderate, and for modality-specific quality metrics, low to moderate, primarily due to residual confounding and imprecision.

The TBS signal we detected (lower values and higher prevalence of degraded TBS in PLWH) is consistent with independent cohorts and extends beyond density per se. In middle-aged HIV-positive men on treatment, Sellier et al. demonstrated disrupted trabecular micro-architecture with lower volumetric trabecular density at both tibia and radius (≈16–17% decrements), consonant with degraded texture metrics and elevated bone fragility risk [31]. Narrative and scoping syntheses focused on “bone quality” in HIV conclude that texture, microarchitecture, and material properties deteriorate in PLWH, with likely contributions from chronic immune activation and antiretroviral exposures—again matching our cross-modality pattern that TBS and HR-pQCT each add information beyond DXA [33].

Tissue-level mechanics by impact microindentation (BMSi) showed HIV-associated decrements in our pooled analysis, and the broader literature supports the clinical relevance of BMSi as a complementary risk signal. While most BMSi studies in HIV remain single-center, general population data and technical reviews indicate that microindentation captures bone material properties not reflected in density or macro-architecture and can discriminate fracture status independent of BMD; this helps contextualize the consistent −3 to −4 BMSi gaps we observed between PLWH and comparators [8]. Taken together with HR-pQCT/TBS, these results argue for a multidimensional framework for skeletal assessment in HIV, particularly in patients whose BMD appears “non-osteoporotic” but who accumulate other quality deficits.

Clinically, the vertebral fracture (VF) burden we observed despite frequently non-osteoporotic BMD is concordant with meta-analytic evidence. Ilha et al. reported that HIV-positive individuals have more than twice the odds of VFs compared with HIV-negative controls, with high between-study heterogeneity reflecting differences in age, treatment era, and imaging methods [34]. Our findings that age and glucocorticoid exposure predict VFs echo the broader HIV literature synthesizing classic risk factors with HIV-specific drivers (e.g., inflammation, ART exposure) [38]. Additionally, vitamin-D insufficiency appears common and clinically meaningful in HIV: in a case–control series of 100 PLWH vs. 100 controls, Atteritano et al. found markedly higher VF prevalence in PLWH and a strong association between low 25-OH-vitamin-D and VFs, underscoring an actionable cofactor that may amplify microarchitectural fragility [35].

Drug-specific patterns provide mechanistic plausibility for our modality-specific signals. Randomized phase-3 trials showed that TAF-based initial regimens preserve BMD substantially better than otherwise-identical TDF-based backbones through 48 weeks (spine −1.30% vs. −2.86%; hip −0.66% vs. −2.95%), without compromising virologic efficacy [36]. Reviews focusing on protease inhibitors similarly implicate PI-containing regimens in greater bone loss and possibly fracture risk, likely via effects on osteoclastogenesis, vitamin D metabolism, and systemic inflammation [37]. These pharmacologic signatures dovetail with our observations that prior TDF and PI exposure tracked with worse cortical indices and lower FE-strength, suggesting that quality-oriented metrics may be especially sensitive to cumulative ART effects.

Finally, emerging proximal-femur imaging using QCT and MRI integrates the story: PLWH exhibit lower trabecular vBMD, poorer trabecular morphology, and higher marrow adiposity at the hip compared to matched controls—changes that imply lower femoral strength even when DXA T-scores are not frankly osteoporotic [39]. Coupled with methodologic data supporting reproducibility of FE-derived strength estimates [32] and narrative syntheses linking HIV pathobiology and ART to bone quality deterioration [33], these results strengthen the case for incorporating TBS and, where available, HR-pQCT/QCT into research protocols and for targeting modifiable risks (vitamin D insufficiency, smoking, glucocorticoids, and TDF/PI exposure when alternatives exist) in clinical care.

In routine HIV care—especially where resources are constrained—a feasibility-first pathway can embed bone health into standard visits without new infrastructure. Begin with structured triage using clinical history (prior low-trauma fracture, falls, and low BMI), medication review (glucocorticoids and ART exposures), and simple labs already common in HIV clinics (vitamin D, where locally available). Provide brief counseling and written prompts targeting modifiable risks (smoking cessation, weight-bearing/impact exercise, and sunlight/dietary strategies for vitamin D/calcium) and schedule reassessment at 12–24 months or sooner after major ART or glucocorticoid changes. Where densitometry is unavailable, apply validated clinical risk tools and contemporaneously review ART to preferentially select bone-sparing options when clinically interchangeable; when densitometry is available, incorporate adjunct measures per local protocols without extending visit length. Task shifting to nurses and pharmacists (checklist-driven “Practice Box”) and integrating order sets into the EHR can standardize delivery with minimal added time.

For service design and quality improvement, we propose explicit referral triggers and audit metrics rather than technology expansion. Referral to specialist evaluation should be prompted by discordant phenotypes (e.g., fragility fracture despite “non-osteoporotic” BMD), unexplained height loss/back pain suggestive of silent vertebral fracture, or repeated falls. Program-level dashboards can track the following: (i) the proportion of eligible patients with documented fracture-risk assessment; (ii) completion of medication review with an ART bone-sparing consideration when appropriate; (iii) delivery of brief lifestyle counseling; and (iv) timely reassessment after treatment changes. Sites can adopt a minimal “bone bundle” (risk checklist, counseling script, order set, follow-up interval) and iteratively PDSA-cycle it for flow efficiency. In parallel, pragmatic research should prioritize implementation outcomes (reach, fidelity, and cost/time per patient) and equity (uptake in high-risk subgroups), ensuring that any added measurements translate into earlier prevention or treatment decisions, not just additional testing.

### 4.2. Strengths and Limitations

Strengths include protocol registration, comprehensive multimodal capture (HR-pQCT/FE, TBS, BMSi, femoral QCT/MRI, VFs), dual independent screening and extraction with good agreement, and explicit risk-of-bias integration with SWiM-based synthesis.

This review also has several limitations that should temper interpretation. First, heterogeneity across studies was substantial in design (cross-sectional vs. longitudinal), populations (age, sex distribution, duration of HIV, and nadir/current CD4), and imaging platforms (HR-pQCT vendors, acquisition protocols, and finite-element pipelines; different TBS software/thresholds), which constrained meta-analytic pooling and likely widened between-study variance. For HR-pQCT, vendor/generation, voxel size, and segmentation/FE pipelines varied across studies; although we harmonized to percent differences and stratified by site and generation, residual technical heterogeneity likely inflated between-study variance. Secondly, most cohorts were from high-income settings with long-term ART exposure, limiting generalizability to resource-limited contexts, ART-naïve individuals, or populations with different comorbidity burdens (tuberculosis and undernutrition). Thirdly, residual confounding is probable: smoking, glucocorticoids, hypogonadism, vitamin D status, and physical activity were inconsistently measured or adjusted for and may partly account for observed gaps in microarchitecture and BMSi. Fourthly, several HR-pQCT reports provided incomplete numerical data for key parameters, and BMSi studies were single-center with modest sample sizes, increasing the risk of imprecision and small-study effects. Fifthly, fracture ascertainment relied predominantly on vertebral fracture assessment or radiographs without systematic adjudication of non-vertebral outcomes, limiting causal inference between “bone quality” metrics and incident, patient-important fractures in PLWH. Finally, publication bias cannot be excluded given the predominance of positive directional findings and the relative novelty of advanced phenotyping modalities in HIV research.

## 5. Conclusions

Across modalities, PLWH demonstrate consistent reductions in cortical and trabecular microarchitecture (HR-pQCT/FE-strength) and tissue-level properties (BMSi), with modest TBS decrements and a clinically relevant vertebral-fracture burden. While biologically plausible and aligning with ART exposure profiles, causality cannot be inferred from observational evidence. In practice, TBS and VFA are scalable adjuncts to refine risk when BMD is not overtly osteoporotic; HR-pQCT/BMSi add mechanistic value in research or complex cases. Prospective and interventional studies linking these measures to incident fractures are needed to translate bone “quality” profiling into reduced fragility in PLWH.

## Figures and Tables

**Table 1:** ROBINS-I summary (non-randomized studies; *n* = 13).

Study (Year)	Design	Overall ROBINS-I Judgment	Brief Rationale
Calmy 2013 [16]	Cross-sectional	Moderate	Between-group confounding (lifestyle, ART history) was partly adjusted; clear outcome measurement.
Biver 2014 [17]	Cross-sectional	Serious	Elderly men on long-term ART; residual confounding/selection was likely; limited adjustment.
Macdonald 2020 [18]	Cross-sectional	Moderate	Adjusted analyses; some residual confounding; complete outcome reporting.
Foreman 2020 [19]	Cross-sectional	Moderate	Multivariable models; potential residual confounding (smoking/PI duration).
Güerri-Fernández 2016 [20]	Cross-sectional	Moderate	BMSi measurement was valid; confounding was partially addressed.
Lerma-Chippirraz 2019 [21]	Longitudinal (pre/post ART)	Moderate	No randomized allocation; time-varying confounding was possible.
Soldado-Folgado 2022 [22]	Cross-sectional	Moderate	Non-progressor phenotype; confounding/selection was addressed incompletely.
Rins-Lozano 2025 [24]	Cross-sectional	Moderate	Group comparison (immunologic responders vs. non-responders) with confounding risk.
McGinty 2019 [25]	Cohort	Moderate	Adjusted for key covariates; remaining confounding was possible.
Sharma 2018 [26]	Cross-sectional	Moderate	Large sample; adjusted; unmeasured confounding was possible.
Guan 2021 [27]	Longitudinal (pre/post ART)	Moderate	No randomization; co-interventions were possible; good outcome measurement.
Llop 2018 [28]	Cross-sectional	Serious	No control group; confounding/selection; outcome classification variability.
Gazzola 2015 [29]	Cross-sectional	Serious	No control group; confounding; variable VF ascertainment.

**Table 2:** RoB 2 summary (randomized/switch evidence; *n* = 1).

Study (Year)	Comparison	Randomization Process	Deviations from Intended Interventions	Missing Outcome Data	Measurement of Outcome	Selection of Reported Result	Overall RoB 2
Soldado-Folgado 2023 [23]	Switch TDF→TAF vs. control (pilot randomized)	Some concerns (small sample; concealment not fully detailed)	Low	Low	Low	Low	Some concerns

**Table 3:** Study characteristics (populations, modality, skeletal site).

Study (Year)	Country/Setting	Design	*n* PLWH	*n* Control	Sex/Age (Mean ± SD or Median[IQR])	Modality	Skeletal Site(s)	ART Status (Key)
Calmy 2013 [16]	Switzerland	Cross-sectional	92	95	Premenopausal women; age 41 ± 8 (PLWH)	HR-pQCT	Distal radius and tibia	On ART; many with TDF
Biver 2014 [17]	Switzerland	Cross-sectional	70	61	Elderly men on successful ART; age ≈ 63	HR-pQCT	Radius and tibia	Long-term ART; PI prevalent
Macdonald 2020 [18]	Canada	Cross-sectional	103	102	Women ~40–60 y	HR-pQCT	Radius and tibia	Varied ART; TDF exposure recorded
Foreman 2020 [19]	USA (UCSF)	Cross-sectional	103	77	Mixed; mean age ~52	HR-pQCT	Radius and tibia	Long-term ART; PI/TDF captured
Guerri-Fernández 2016 [20]	Spain	Cross-sectional	85	79	Middle-aged; both sexes	Microindentation (BMSi)	Tibial midshaft	Many on ART
Lerma-Chippirraz 2019 [21]	Spain	Longitudinal	44	—	PLWH starting ART	BMSi	Tibia	ART initiation
Soldado-Folgado 2022 [22]	Spain	Longitudinal	59	—	Adults on TDF	BMSi	Tibia	Switch TDF→TAF
Soldado-Folgado 2023 [23]	Spain	Cross-sectional	85	60	Both sexes	BMSi	Tibia	Mixed ART
Rins-Lozano 2025 [24]	Spain	Cross-sectional	82	—	Immunologic non-responders vs. responders	BMSi	Tibia	Suppressed VL; differing CD4
McGinty 2019 [25]	Ireland	Cohort	201	262	Mixed; adults	TBS	Lumbar spine (DXA-derived)	ART mixed; PI/TDF data
Sharma 2018 [26]	USA	Cross-sectional	319	118	Women	TBS	Lumbar spine	ART mixed
Guan 2021 [27]	China	Longitudinal	233	—	Adults, mean 36.6 ± 11.1	TBS + BMD	Lumbar spine	Pre-ART → 48 wks ART
Llop 2018 [28]	Spain	Cross-sectional	199	—	Adults	VFA (X-ray)	Thoracolumbar	ART mixed
Gazzola 2015 [29]	Italy	Cross-sectional	194	—	Adults	Spine X-ray VFs	Thoracolumbar	ART mixed

PLWH, people living with HIV; HR-pQCT, high-resolution peripheral quantitative computed tomography; DXA, dual-energy X-ray absorptiometry; BMSi, Bone Material Strength index; TBS, trabecular bone score; VFA, vertebral fracture assessment; ART, antiretroviral therapy; TDF, tenofovir disoproxil fumarate; TAF, tenofovir alafenamide; PI, protease inhibitor.

**Table 4:** Microarchitecture and Tissue Quality.

Study	Metric(s) (Site)	PLWH (Mean ± SD)	Controls (Mean ± SD)	Between-Group Difference/Effect
Calmy 2013 [16]	Failure load (FEA, radius/tibia)	↓ vs. controls (exact means NR)	—	Lower estimated failure load; lower Tb.N, higher Tb.Sp, thinner Ct.Th in PLWH; several ~5–15% differences were reported in text/figures.
Biver 2014 [17]	HR-pQCT: Tb.N, Tb.Sp, Ct.Th	NR	NR	Microstructural alterations (trabecular and cortical) were observed in elderly men on ART vs. controls (directionally worse in PLWH).
Macdonald 2020 [18]	Failure load (radius/tibia)	Lower vs. controls (NR exact)	—	PLWH reduced failure load and cortical measures; associations with TDF history were noted.
Foreman 2020 [19]	HR-pQCT comprehensive	NR	NR	PLWH showed worse trabecular/cortical indices; PI exposure and smoking were associated with lower failure load/stiffness.
Guerri-Fernández 2016 [20]	BMSi (tibia)	77.2 ± 6.9	80.6 ± 6.2	−3.4 units (*p* < 0.01)—lower tissue-level strength in PLWH.
Lerma-Chippirraz 2019 [21]	BMSi change after ART start	−2.1 ± NR at 24–48 wks	—	Early decline after ART initiation (*p* = 0.02).
Soldado-Folgado 2022 [22]	BMSi (TDF→TAF)	+2.5 units (6–12 mo post-switch)	—	Significant BMSi improvement following TAF switch.
Soldado-Folgado 2023 [23]	BMSi	78.4 ± 7.1	82.0 ± 6.4	−3.6 (*p* < 0.01) cross-sectional gap.
Rins-Lozano 2025 [24]	BMSi	INR 76.7 ± 6.3 vs. IR 80.2 ± 6.1	—	−3.5 units in immunologic non-responders (*p* = 0.001).
McGinty 2019 [25]	TBS (median [IQR])	1.349 [1.263–1.436]	1.380 [1.301–1.453]	−0.031 (*p* = 0.009) unadjusted; adj. β for HIV −0.037 (*p* = 0.002).
Sharma 2018 [26]	Degraded TBS (<1.35)	Higher prevalence	—	HIV+ women were 64% more likely to have degraded TBS (adj).
Guan 2021 [27]	TBS at baseline → 48 wks	Decline early, then partial recovery	—	19.3% had normal BMD but abnormal TBS pre-ART.
Llop 2018 [28]	Asymptomatic VF prevalence	~25% (varied by age)	—	Subclinical VF was frequent despite non-osteoporotic BMD in many.
Gazzola 2015 [29]	VF prevalence	12.4% (24/194)	—	Predictors: age (aOR 1.09/yr); steroids (aOR 3.64); many VFs with non-osteoporotic BMD.

FEA, finite-element analysis; Tb.N, trabecular number; Tb.Sp, trabecular separation; Ct.Th, cortical thickness; BMSi, Bone Material Strength index; TBS, trabecular bone score; VF, vertebral fracture; aOR, adjusted odds ratio; PI, protease inhibitor; TDF, tenofovir disoproxil fumarate; TAF, tenofovir alafenamide; Δ, change/difference.

**Table 5:** Predictors and longitudinal effects.

Study	Predictor/Exposure	Outcome	Effect (β/OR/HR/Δ)
Foreman 2020 [19]	Protease inhibitor exposure; current smoking	HR-pQCT failure load/stiffness	Lower strength indices with PI and smoking (multivariable).
Macdonald 2020 [18]	TDF history	HR-pQCT failure load/cortical	Prior TDF were associated with worse cortical indices (directional).
McGinty 2019 [25]	HIV status (vs. −)	TBS	Adj. β −0.037 (*p* = 0.002); attenuates with smoking; in PLWH, PI exposure ↓TBS, lower nadir CD4 ↓TBS.
Sharma 2018 [26]	HIV+ (women)	Degraded TBS	+64% likelihood of degraded TBS vs. HIV− after adjustment.
Guan 2021 (233) [27]	ART initiation	TBS and BMD	Early decline in TBS/BMD post-ART, then partial recovery by week 48; 19.3% had normal BMD but abnormal TBS pre-ART.
Guerri-Fernández 2016 [20]	HIV+ vs. −	BMSi	−3.4 units (*p* < 0.01).
Lerma-Chippirraz 2019 [21]	ART start	BMSi	−2.1 units by ~6–12 mo (*p* = 0.02).
Soldado-Folgado 2022 [22]	Switch TDF→TAF	BMSi change	+2.5 units (*p* < 0.05) post-switch.
Rins-Lozano 2025 [24]	Immunologic non-response	BMSi	−3.5 units vs. responders (*p* = 0.001).
Llop 2018 [28]	Age; steroids	Asymptomatic VFs	Older age ↑ VFs; steroid use ↑ VFs (multivariable).
Gazzola 2015 [29]	Age; steroids	VFs	aOR 1.09/yr; aOR 3.64 for steroids; 70% VFs at non-osteoporotic BMD. PubMed.
Soldado-Folgado 2023 [23]	HIV+ vs. −	BMSi	−3.6 units (*p* < 0.01).
Biver 2014 [17]	Long-term ART	HR-pQCT	Altered trabecular and cortical microarchitecture vs. controls.
Calmy 2013 [16]	HIV+/ART	HR-pQCT	Lower Tb.N, Ct.Th; higher Tb.Sp; lower failure load vs. controls.

OR/HR, odds/hazard ratio; aOR, adjusted odds ratio; β, regression coefficient; TBS, trabecular bone score; BMD, bone mineral density; BMSi, Bone Material Strength index; HR-pQCT, high-resolution peripheral quantitative computed tomography; PI, protease inhibitor; TDF, tenofovir disoproxil fumarate; TAF, tenofovir alafenamide; VF, vertebral fracture; CD4, CD4+ T-cell count.

## Data Availability

Not applicable.
